# Proportional Frequency and Clinical Characteristics of Gastric, Esophageal, and Gastroesophageal Junction Cancers in a Private Medical Group in Brazil

**DOI:** 10.14740/wjon2551

**Published:** 2025-12-17

**Authors:** Gustavo Fernandes, Felipe D’Almeida Costa, Flavia Paiva Proenca Lobo Lopes, Cristovam Scapulatempo-Neto, Giovanni Bomfim, Sarah Goncalves, Thiago Martins, Roberto Soler

**Affiliations:** a Department of Oncology, Dasa, Sao Paulo - SP, 06455-010, Brazil; b Department of Pathology, Dasa, Sao Paulo - SP, 06455-010, Brazil; c Department of Health Research, Dasa, Sao Paulo - SP, 06455-010, Brazil; d Department of Pathology and Genomics, Dasa, Sao Paulo - SP, 06455-010, Brazil; e Department of Medical Affairs, Astellas Farma Brasil, Sao Paulo - SP, 04730-090, Brazil

**Keywords:** Adenocarcinoma, Biomarker, Brazil, Cancer, Esophageal, Gastric, Gastroesophageal junction, Proportional frequency

## Abstract

**Background:**

Gastric cancer (GC) and esophageal cancer (EC) are among the most prevalent malignancies globally and are leading causes of cancer-related mortality. Gastroesophageal junction cancer (GEJC) is considered a distinct entity with specific histopathological characteristics. This study aimed to determine the proportional frequency of GEJC; the clinicopathological characteristics of GC, EC, and GEJC; and adherence to clinical diagnostic guidelines using data from a large private healthcare network in Brazil.

**Methods:**

In this retrospective, cross-sectional, descriptive study and database review, records from a Brazilian medical group (Dasa) were evaluated for adults (aged ≥ 18 years) who underwent upper digestive endoscopy between July 1, 2019, and June 30, 2022. Test results from the biopsy date associated with GC, EC, or GEJC diagnosis through December 2022 were collected retrospectively.

**Results:**

In total, 181 patients were evaluated, including 116 (64.1%) with GC, 37 (20.4%) with EC, 22 (12.2%) with GEJC, and six (3.3%) with lesions in ≥ 2 locations of interest. The ratio of GEJC to GC cases was 0.190, and the ratio of GEJC to EC cases was 0.595. Median (interquartile range) age at diagnosis was 67 (60 - 76) years, and most patients (57.5%) were aged 60 - 79 years. Sixty-two (53.4%), 27 (73.0%), 17 (77.3%), and four (66.7%) patients with GC, EC, GEJC, and lesions in ≥ 2 locations of interest, respectively, were male. The most frequently observed Lauren subtype was intestinal type for both GC and GEJC (68 (58.6%) patients and 18 (81.8%) patients, respectively). Adenocarcinoma was the most frequent histologic subtype observed in patients with EC (n = 21; 56.8%). Among patients with GC, EC, or GEJC and immunohistochemical biomarker testing, human epidermal growth factor receptor 2 expression was positive in 2/28 (7.1%), 1/3 (33.3%), and 1/2 (50.0%) patients, respectively, and mismatch repair deficiency was detected in 2/28 (7.1%), 0/3 (0%), and 0/2 (0%) patients. No patients with GEJC were tested for programmed cell death ligand 1 or claudin 18.2.

**Conclusions:**

An improved understanding of GEJC characteristics in Brazil is essential for facilitating early diagnosis, identifying optimal treatment strategies, and informing public health policies. The extremely low rates of biomarker testing in this study revealed a significant gap in the implementation of modern oncology guidelines.

## Introduction

Cancers of the stomach and esophagus are among the most prevalent malignancies globally, ranking fifth and 11th, respectively, and are a leading cause of cancer-related mortality [1]. There were more than 1.4 million new cases of gastric cancer (GC) and esophageal cancer (EC) worldwide in 2022 [1]. Gastroesophageal junction cancer (GEJC) is characterized by highly aggressive tumors that originate adjacent to the gastroesophageal junction (GEJ), with an epicenter located 1 cm above to 2 cm below the cardia [2-4]. GEJC is considered a distinct entity with specific histopathological characteristics [2, 4].

Traditionally, GC has exhibited higher incidence rates than EC and GEJC; however, recent data indicate declining GC incidence and increasing incidence of EC and GEJC [5, 6]. A population-based study in the United States revealed an incidence of 1.40 per 100,000 individuals for EC and 0.83 per 100,000 individuals for GEJC during 1976 - 2019, with increases during the 1990s and early 2000s [7]. An analysis of the Surveillance, Epidemiology, and End Results (SEER) cancer registry program in the United States demonstrated an approximately 2.5-fold increase in the incidence of GEJC during 1973 - 1992 [5]. Similar findings have emerged from studies in Europe, Canada, and Japan [8-12]. The decline in GC incidence is largely attributed to the management of prevalent risk factors and advancements in prevention of *Helicobacter pylori* (*H. pylori*) infection, which promotes the development of GC [6, 13]. The observed rise in GEJC incidence may be influenced by increasing prevalence of obesity and Barrett’s esophagus; other established risk factors include smoking, alcohol consumption, gastrointestinal reflux disease, and high fat intake [2, 5, 14]. Unlike in GC, *H. pylori* infection is not considered to be a risk factor for GEJC and may even be protective against the development of GEJC [2, 5].

Robust epidemiological data on the incidence and prevalence of GEJC in developing countries remain limited [1]. In Brazil, for the 3-year period spanning 2023 - 2025, the estimated number of new cases of GC is 9.94 per 100,000 inhabitants, and the estimated number of new cases of EC is 5.07 per 100,000 inhabitants [15]. In 2020, there were 6.54 deaths from GC per 100,000 Brazilians and 3.92 deaths from EC per 100,000 Brazilians [15]. A lack of comprehensive population studies on GEJC within the Brazilian population is a significant gap, as accurate diagnosis and proper staging are critical to address patients’ individual needs, and GEJC treatment is developing quickly.

Primary classification, subclassification, identification of biological markers, and tumor staging are pivotal factors influencing treatment response. Distinct genomic profiles and histopathological features characterize tumors in the esophagus, stomach, and GEJ, necessitating accurate tumor characterization. Immunohistochemistry (IHC) is increasingly employed to identify specific biomarkers that can be used to predict the efficacy of novel therapies based on the presence of these biomarkers, such as human epidermal growth factor receptor 2 (HER2) overexpression, programmed cell death ligand 1 (PD-L1) expression, mismatch repair (MMR)/microsatellite instability (MSI) status, or claudin 18.2 (CLDN18.2) expression [16-19]. Approximately 15-20% of GEJC cases reportedly overexpress HER2, and overexpression is more common in those with intestinal histology [16, 17]. PD-L1 is not normally expressed in gastric tissue but is expressed in 30-65% of gastric tumors [20], and MSI has been reported in 6-24% of GEJ adenocarcinoma [21]. CLDN18.2 expression was positive (defined as ≥ 75% of tumor cells showing moderate to strong membranous claudin 18 (CLDN18) staining) in 38% of patients with locally advanced unresectable or metastatic gastric or GEJ adenocarcinoma screened for participation in clinical trials [22, 23]. The anti-programmed cell death protein 1 antibodies pembrolizumab and nivolumab are targeted therapies approved for the treatment of GEJC. The CLDN18.2-targeted antibody zolbetuximab is approved for the treatment of locally advanced unresectable or metastatic HER2-negative gastric or GEJ adenocarcinoma in adults with CLDN18.2-positive tumors.

This study aimed to determine the following using data from a large private healthcare network in Brazil: the proportional frequency of GEJC; the clinicopathological characteristics of GC, EC, and GEJC; and secondarily, adherence to clinical diagnostic guidelines.

## Materials and Methods

### Study design

This was a retrospective, cross-sectional, descriptive study and database review based on records from a Brazilian medical group (Dasa), including upper digestive endoscopy (UDE), biopsy, and IHC biomarker findings. Records were evaluated from patients who were aged ≥ 18 years, underwent UDE at Dasa diagnostic units and hospitals in Brazil between July 1, 2019, and June 30, 2022, and had pathological evaluation of UDE biopsy and a UDE report. Test results from the date of UDE through December 2022 were collected retrospectively. The index biopsy was the biopsy associated with the cancer diagnosis (or, in cases involving multiple biopsies, the first biopsy associated with the cancer diagnosis). The following data were recorded: demographic details including age at diagnosis (based on UDE date), sex, and geographical location (Brazilian state) in which UDE was performed, and biopsy data including *H. pylori* status (detected by histochemical staining), Lauren classification (for GC and GEJC), histologic classification (for EC), and IHC biomarker status (including HER2, PD-L1 combined positive score, and MSI/MMR) when available (for all tumors).

The Lauren classification categorizes GC and GEJC into three primary types: intestinal type (comprising, in this study, not otherwise specified adenocarcinoma, tubular adenocarcinoma, and mucinous adenocarcinoma with an intestinal phenotype), diffuse type (comprising poorly cohesive carcinoma in this study), and mixed type.

EC was histologically classified as squamous cell carcinoma, small cell carcinoma, or adenocarcinoma.

Tumors whose epicenter was between 1 cm above and 2 cm below the gastric cardia, corresponding to Siewert type II classification [3, 4, 24], were classified as GEJC.

### Endpoints

The primary endpoints were rates of GC, EC, and GEJC diagnosed by UDE biopsy; rate of GEJC relative to GC; and rate of GEJC relative to EC. Secondary endpoints included descriptive histopathologic characteristics of GC and GEJC (Lauren classification and *H. pylori* status) and rates of biomarker testing in patients with GC, EC, GEJC, or lesions in two or more sites of interest.

Adherence to clinical diagnosis guidelines was assessed by evaluating the rate of biomarker testing, including HER2 and MMR status. The rate was calculated by determining the percentage of patients with HER2 and MMR test results across the overall study population and within each cancer type (GC, EC, and GEJC). These specific biomarkers were evaluated because data for other relevant biomarkers were not widely available during the study period.

### Statistical analysis

All data were anonymized and encrypted. To minimize bias, patients with tumors in > 1 site (GC, EC, and/or GEJC) were not included in rate calculations. Data are presented as median (interquartile range (IQR)) for continuous measures and n (%) for categorical measures. For continuous variables, P values were obtained using the Kruskal-Wallis test. For categorical variables, P values were obtained using the Chi-square test. An alpha value of 0.05 for statistical significance was applied.

### Ethical conduct of the study

This study was conducted in accordance with the ethical principles of the Declaration of Helsinki and Good Clinical Practice, and in compliance with all applicable requirements in Brazil ensuring the rights of participants in non-interventional studies. Institutional review board approval (CAEE: 68599823.0.0000.5455) was obtained. Data extraction was performed in compliance with privacy policy and the General Data Protection Law in Brazil. Written informed consent was waived for this study given its retrospective design and anonymized data.

## Results

### Patients

**Figure 1:**
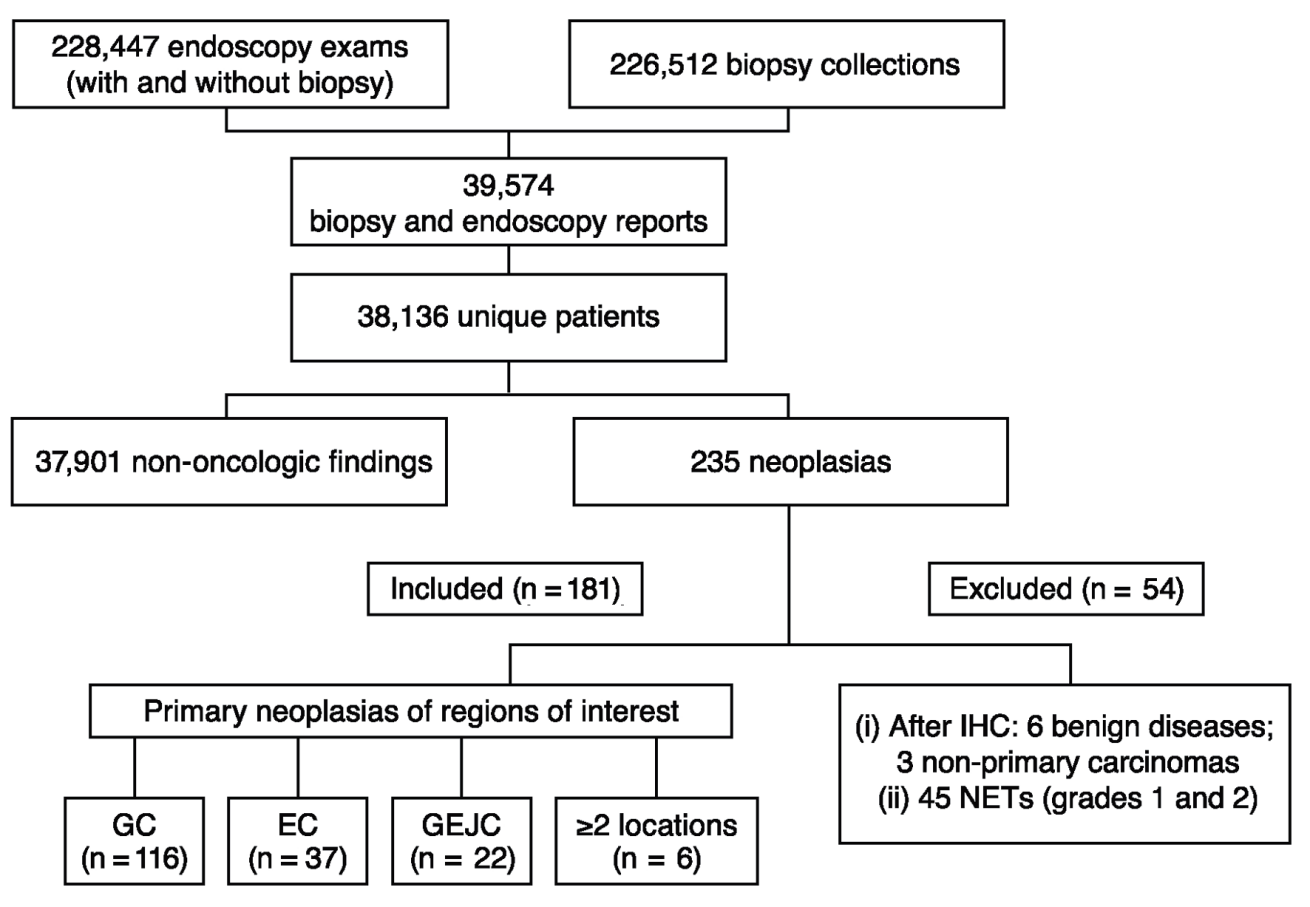
Flowchart of data extraction and filtering. EC: esophageal cancer; GC: gastric cancer; GEJC: gastroesophageal junction cancer; IHC: immunohistochemistry; NET: neuroendocrine tumor.

A total of 228,447 UDE and 226,512 biopsy reports were identified in the Dasa database (Fig. 1). Cross-referencing the unique patient identifier numbers revealed 39,574 reports from 38,136 unique patients. Among these unique patients, 235 were initially identified as having malignant neoplasia according to the biopsy results; after evaluating IHC biomarkers, 54 patients were excluded due to a final diagnosis of benign disease (n = 6), metastatic lesions from non-gastric primary carcinomas (n = 3), or neuroendocrine tumors (NETs; grade 1 or 2, n = 45). Thus, 181 patients were evaluated in the study.

### Rates of GC, EC, and GEJC

Of the 181 patients evaluated, 116 (64.1%) were diagnosed with GC, 37 (20.4%) were diagnosed with EC, 22 (12.2%) were diagnosed with GEJC, and six (3.3%) had lesions in ≥2 locations of interest. The rates of GC, EC, and GEJC did not exclude squamous cell carcinoma. The ratio of GEJC to GC cases was 0.190, and the ratio of GEJC to EC cases was 0.595.

### Demographic characteristics

Median (IQR) age at diagnosis was 67 (60 - 76) years across all 181 patients evaluated and was similar across all four groups (patients with GC, EC, GEJC, and lesions in ≥ 2 locations of interest; Table 1). Across all four groups, the proportional frequency of cases was highest among patients aged 60 - 79 years (57.5% of patients in the study) and lowest among those aged < 50 years (11.6%). Approximately half (53.4%) of patients with GC were male, whereas approximately three-quarters of patients with EC (73.0%) and GEJC (77.3%) were male. The sex ratio did not differ significantly across the four groups (P = 0.057). Overall, most patients (93.9%) were from the state of Sao Paulo.

**Table 1:** Characteristics of the Study Population

Characteristic	GC (n = 116)	EC (n = 37)	GEJC (n = 22)	≥ 2 locations (n = 6)	P value
Age at diagnosis, years, median (IQR)	72 (60 - 79)	65 (59 - 74)	65 (59 - 76)	64 (62 - 65)	0.200^a^
Age category at diagnosis, n (%)					0.120^a^
< 40 years	8 (6.9)	0 (0)	1 (4.5)	0 (0)	
40 - 49 years	4 (3.4)	5 (13.5)	2 (9.1)	1 (16.7)	
50 - 59 years	16 (13.8)	5 (13.5)	3 (13.6)	0 (0)	
60 - 69 years	27 (23.3)	10 (27.0)	7 (31.8)	4 (66.7)	
70 - 79 years	34 (29.3)	15 (40.5)	6 (27.3)	1 (16.7)	
≥ 80 years	27 (23.3)	2 (5.4)	3 (13.6)	0 (0)	
Sex, n (%)					0.057^b^
Male	62 (53.4)	27 (73.0)	17 (77.3)	4 (66.7)	
Female	54 (46.6)	10 (27.0)	5 (22.7)	2 (33.3)	
Geographic region (state), n (%)					0.600^b^
Sao Paulo	108 (93.1)	36 (97.3)	21 (95.5)	5 (83.3)	
Rio de Janeiro	2 (1.7)	1 (2.7)	1 (4.5)	0 (0)	
Goias	1 (0.9)	0 (0)	0 (0)	0 (0)	
Distrito Federal	5 (4.3)	0 (0)	0 (0)	1 (16.7)	
*Helicobacter pylori* status, n (%)					0.059^b^
Positive	13 (11.2)	3 (8.1)	3 (13.6)	0 (0)	
Negative	39 (33.6)	5 (13.5)	2 (9.1)	1 (16.7)	
Not performed	64 (55.2)	29 (78.4)	17 (77.3)	5 (83.3)	
Biomarker (HER2, MMR/MSI) evaluation performed, n (%)	28 (24.1)	3 (8.1)	2 (9.1)	0 (0)	0.065^b^

^a^For continuous variables, P values were obtained using the Kruskal-Wallis test. ^b^For categorical variables, P values were obtained using the Chi-square test. EC: esophageal cancer; GC: gastric cancer; GEJC: gastroesophageal junction cancer; HER2: human epidermal growth factor receptor 2; IQR: interquartile range; MMR: mismatch repair; MSI: microsatellite instability.

### Histopathologic characteristics

Among 181 patients diagnosed with primary GC, EC, or GEJC, or lesions in ≥ 2 locations of interest, 66 (36.5%) underwent *H. pylori* testing (Table 1). Fifty-two patients with GC, eight patients with EC, five patients with GEJC, and one patient who had lesions in ≥ 2 locations of interest were tested. Among these patients, 13 of 52 (25.0%), three of eight (37.5%), three of five (60.0%), and zero of one (0%), respectively, were *H. pylori* - positive.

Tubular adenocarcinoma was the most frequent histologic subtype, observed in 57 (49.1%) and 16 (72.7%) patients with GC and GEJC, respectively. The most frequently observed Lauren subtype was intestinal type for both GC and GEJC (68 (58.6%) patients and 18 (81.8%) patients, respectively; Table 2).

**Table 2:** Distribution of GC and GEJC by Lauren Classification

Lauren classification, n (%)	GC (n = 116)	GEJC (n = 22)	≥ 2 locations (n = 6)
Intestinal type^a^	68 (58.6)	18 (81.8)	4 (66.7)
Diffuse type^b^	34 (29.3)	2 (9.1)	1 (16.7)
Mixed type	14 (12.1)	2 (9.1)	1 (16.7)

^a^Intestinal type included NOS adenocarcinoma, tubular adenocarcinoma, and mucinous adenocarcinoma with intestinal phenotype. ^b^Diffuse type included only poorly cohesive carcinoma. GC: gastric cancer; GEJC: gastroesophageal junction cancer; NOS: not otherwise specified.

Adenocarcinoma was the most frequent histologic subtype observed in patients with EC (n = 21; 56.8%; Table 3).

**Table 3:** Distribution of EC by Tumor Histologic Type

Histologic type, n (%)	EC (n = 37)
Adenocarcinoma	21 (56.8)
Squamous cell carcinoma	15 (40.5)
Small cell carcinoma	1 (2.7)

EC: esophageal cancer.

### IHC biomarkers

IHC biomarkers (HER2 and MMR/MSI) were assessed in 33 (18.9%) of the 175 patients diagnosed with primary GC, EC, or GEJC (Tables 1 and 4), and these cases were considered adherent to clinical guidelines. Biomarker evaluation occurred more frequently in patients with GC (24.1%) compared with EC (8.1%) and GEJC (9.1%; P = 0.065; Table 1).

**Table 4:** IHC Biomarkers in the Study Population

Diagnosis	Total patients, n	HER2		MMR	
		Patients tested, n (%)	HER2-positive^a^, n (%)	Patients tested, n (%)	MMR deficient^a^, n (%)
GC	116	28^b^ (24.1)	2 (7.1)	28^c^ (24.1)	2 (7.1)
EC	37	3 (8.1)	1 (33.3)	3 (8.1)	0 (0)
GEJC	22	2 (9.1)	1 (50.0)	2 (9.1)	0 (0)

^a^Among patients tested. ^b^For one case, a fluorescence *in situ* hybridization test was not performed at the institution, so the results were considered uncertain. ^c^For one case, a retest for an equivocal result was not performed at the institution, so the results were considered uncertain. EC: esophageal cancer; GC: gastric cancer; GEJC: gastroesophageal junction cancer; HER2: human epidermal growth factor receptor 2; IHC: immunohistochemistry; MMR: mismatch repair.

Two patients with IHC biomarker testing were in the GEJC group, and both had tubular adenocarcinoma. They were evaluated for HER2 status, revealing one positive result; both were tested for MSI/MMR and showed proficient MMR, and one underwent testing for p53 protein, which showed aberrant expression.

No patients with GEJC were tested for PD-L1 or CLDN18.2. In addition, because Epstein-Barr virus (EBV) testing is not routinely performed, EBV status was not available in the database.

## Discussion

GEJC is a growing public health concern; while rare, its incidence is on the rise. Considering the similarities among GC, EC, and GEJC, improved understanding of GEJC is essential to increase diagnostic accuracy, optimize treatment strategies, and enhance patient outcomes. To our knowledge, this study is the first to characterize the proportional frequency of GEJC in a Brazilian real-world setting using data from a private healthcare network.

Results of this study show that the proportional frequency of GC (observed in 64.1% of patients) is higher than that of EC (20.4%) and GEJC (12.2%), consistent with previous literature [5, 6]. However, as the global incidence of GC is declining and the global incidence of GEJC is increasing [5-12], GEJC is a cause for increasing concern. The present findings reveal a GEJC to GC case ratio of 0.190 and a GEJC to EC case ratio of 0.595.

Risk factors for development of GEJC include advanced age and male sex, as well as Barrett’s esophagus, chronic gastroesophageal reflux, obesity, smoking, alcohol consumption, and high fat intake [2, 5]. In the present study, the proportional frequency of GEJC increased with age and was highest in patients aged 60 - 79 years. Most (77.3%) patients with GEJC were male, consistent with studies reporting that rates of GEJC are considerably higher in men compared with women [5, 7].

*H. pylori* infection contributes to the development of GC but may actually be protective against GEJC [2, 5]. In a seminal study, 84.4% of patients with GC were positive for *H. pylori* (odds ratio vs. controls, 3.6 (95% confidence interval, 1.8 - 7.3)) and 63.0% of patients with GEJC were positive for *H. pylori* (odds ratio vs. controls, 0.8 (95% confidence interval, 0.3 - 2.1)) [13]. Therefore, screening for *H. pylori* infection and treatment of positive cases is recommended for GC prevention [25, 26]. However, *H. pylori* results were available for only 66 participants in this study. *H. pylori* status was positive in three of five (60.0%) patients with GEJC who were tested and three of eight (37.5%) patients with EC who were tested. The rate for GEJC is consistent with previous findings [13]. *H. pylori* status was positive in only 13 of 52 (25.0%) patients with GC who were tested. The unexpectedly low rate compared with previous findings [13] may stem from limitations in the testing method (histochemical staining), such as histochemical staining during UDE, influence of inflammatory conditions, alcohol consumption, active bleeding, or medications (e.g., proton-pump inhibitors or antibiotics) [27], or from the low testing rate. Caution is warranted in interpreting the *H. pylori* results in this study due to the limited number of patients tested and lack of information on physicians’ rationale for testing or not testing for *H. pylori*. In Brazil, a daily practice observation is that endoscopists often perform a tumor biopsy on patients with GC or GEJC and do not biopsy the surrounding healthy tissue to look for *H. pylori*. Therefore, biopsies for *H. pylori* were likely performed due to a specific request from the referring physician, as most health insurance providers only reimburse for tests and procedures that are explicitly prescribed. This practice likely resulted in the low number of tested patients within our cohort, as the test is not consistently prescribed by all general practitioners. Due to the selection bias in testing and the small sample size within each subgroup, our findings on *H. pylori* positivity are descriptive only; no definitive conclusions can be drawn.

Despite the global prevalence and mortality associated with malignant tumors of the stomach, esophagus, and GEJ, adherence to diagnostic and treatment guidelines is not well documented in the literature. This lack of compliance may contribute to the relatively high mortality rates observed for these cancers.

Distinct genomic profiles and histopathological features characterize tumors in the esophagus, stomach, and GEJ, necessitating accurate tumor characterization [28]. Consistent use of a classification system that clearly defines GEJC anatomically is critical. Primary classification, subclassification, tumor stage, and biological markers influence treatment response [28, 29]. Most GC and GEJC tumors are adenocarcinoma [29, 30]. In line with this, in the current study, 72.7% of GEJC cases (n = 16) were tubular adenocarcinoma.

Due to the increasing availability of targeted therapies, IHC has become a crucial tool in addition to standard histopathological evaluation. It is now widely used to identify specific biomarkers such as HER2 overexpression, PD-L1 expression, MSI/MMR status, and CLDN18.2 expression, which are essential for guiding the application of novel therapeutic strategies [19, 28]. Approximately 15-20% of GEJC cases reportedly overexpress HER2, and overexpression is more frequent in those with intestinal histology [16, 17]. Current consensus guidelines of the Brazilian Group of Gastrointestinal Tumours recommend evaluating HER2 status, MMR/MSI, PD-L1, and neurotrophic tyrosine receptor kinase in GEJC biopsies [31]. Due to regulatory constraints, certain targeted treatments, such as pembrolizumab and zolbetuximab, received approval in Brazil only recently (at the end of 2023 and this year (2025), respectively). Consequently, routine screening for PD-L1 and CLDN18.2 expression has only recently been implemented. In this study, only two GEJC patients were tested for IHC biomarkers. Both patients underwent HER2 and MSI/MMR testing; HER2 was overexpressed in one (50%), and both (100%) showed proficient MMR. No patients with GEJC were tested for PD-L1 or CLDN18.2 in this study.

The low rate of IHC biomarker testing observed in our study raises significant concerns and appears to stem from three primary barriers: lack of awareness of and adherence to guidelines by physicians, fragmented patient care, and restrictive reimbursement policies in Brazil. The first of these barriers, the inconsistent request for key biomarker tests by attending physicians, may compromise the optimal treatment approach for patients and could be attributed to a lack of awareness of or a failure to adhere to established consensus guidelines, particularly among non-specialist general practitioners. A recent Italian study investigated guideline adherence for the diagnosis, staging, and treatment of GC from the perspectives of both surgeons and patients [32]. Although the study did not specifically address biomarkers or *H. pylori* testing, it highlighted significant discrepancies between surgical practices and guideline recommendations. These findings underscore the need for greater awareness across various aspects of patient management to improve overall guideline adherence. The second issue is the fragmentation of the patient journey. When patients receive care across different healthcare networks, it can lead to data loss and a lack of comprehensive care, hindering the continuity required for a complete diagnostic and treatment pathway. This likely contributed to the low rate of biomarker testing observed in our study, as some patients may have received biomarker testing at another facility. Third, the reimbursement of biomarker testing and targeted therapies in Brazil is strictly regulated. Currently, only HER2 and MMR are being tested in regular IHC panels for all GC and GEJC. PD-L1 testing is reimbursed, but most cases are tested using patient support programs because of high costs and delayed approval from health insurance companies. Zolbetuximab was recently approved for the treatment of GC or GEJC with CLDN18.2 positivity (moderate to strong membranous CLDN18 expression in ≥ 75% of tumor cells) in Brazil. Because the drug was not approved using a companion diagnostic test, laboratories may use validated in-house concentrated antibodies against CLDN18.2, reducing the total cost of the biomarker test. Moreover, there is a patient support program available for CLDN18.2 testing. However, the National Supplementary Health Agency (ANS) currently does not approve the coverage of zolbetuximab for all health insurance plans, reducing access to zolbetuximab for this population. Although the PD-L1 biomarker test itself is reimbursed for GC and GEJC because it is recognized by the ANS, the associated targeted treatment only became covered by health insurance providers in November 2023.

The growing body of evidence from major clinical trials highlights the critical importance of a multi-biomarker testing strategy, a practice that is currently hindered by the barriers discussed above. The KEYNOTE-811 trial demonstrated that adding pembrolizumab to a standard regimen of trastuzumab and chemotherapy significantly improved the objective response rate in patients with HER2-positive advanced GC or GEJC [33]. The benefit was particularly pronounced in patients whose tumors had a PD-L1 combined positive score ≥ 1 [33]. This highlights the necessity of comprehensive diagnostic evaluation for both HER2 and PD-L1 status to identify patients who are most likely to benefit. Given the relatively recent approval of pembrolizumab in Brazil, we anticipate the generation of a robust dataset for pembrolizumab in Brazil in the near future. The CheckMate 649 trial established nivolumab plus chemotherapy as a new standard of care in the first-line setting for patients with HER2-negative, advanced gastric, GEJ, or esophageal adenocarcinoma [34]. The study showed a statistically and clinically significant improvement in progression-free survival (PFS) and overall survival (OS), especially in patients whose tumors expressed PD-L1 with a combined positive score ≥ 5. This reinforces the shift toward a personalized, biomarker-driven approach in cancer management. The SPOTLIGHT [23] and GLOW [22] trials have established zolbetuximab plus chemotherapy as a new standard of care for patients with previously untreated CLDN18.2-positive, HER2-negative, locally advanced unresectable or metastatic gastric or GEJ tumors. These landmark studies showed significant improvements in PFS and OS with zolbetuximab plus chemotherapy compared with placebo plus chemotherapy, providing compelling evidence that routine CLDN18.2 testing is essential for identifying patients who can benefit from this highly effective targeted therapy, which was recently approved in Brazil. This extensive body of global evidence supporting the biomarker-informed approach to cancer treatment highlights the critical need to overcome the local barriers to biomarker testing identified in our study.

Improving care for patients with GC, EC, and GEJC requires a multi-faceted approach. First, there is a need for enhanced physician education on the importance of biomarkers in guiding treatment decisions. Simultaneously, patient care pathways must be improved to ensure a continuous, integrated, and cohesive treatment journey. Additionally, it is crucial to conduct and publish studies reporting biomarker testing rates in Brazil, especially given the current low rates. The data from such research would serve to highlight the clinical value of these tests for patients and to raise awareness among Brazilian authorities.

This study has strengths. It provides a snapshot of real-world diagnostic practices in a Brazilian private healthcare network, yielding results comparable to those of studies conducted in other countries and highlighting the very low testing rate for key biomarkers. It is also the first study evaluating real-world data on the proportional frequency of GEJC in a large cohort from one of the largest private healthcare networks in Brazil, which is significant considering that the bi-/triannual reports of the Brazilian National Cancer Institute do not include GEJC incidence [15].

This study also has limitations. First, like most other database or chart review studies, it is limited by its retrospective design and the potential for missing data. Second, as most (93.9%) of the cases were from one Brazilian state (Sao Paulo) and all were from a single private medical group (Dasa), the results may not be generalizable to patients from other states or institutions. Finally, there was little data available on crucial biomarkers, as extensively discussed.

### Conclusion

This study aimed to determine the proportional frequency of GEJC; the clinicopathological characteristics of GC, EC, and GEJC; and adherence to clinical diagnostic guidelines using data from a large private healthcare network in Brazil. There are several main findings. The ratio of GEJC to GC cases was 0.190, and the ratio of GEJC to EC cases was 0.595. The proportional frequency of GEJC was higher in men (77.3% of patients with GEJC) than in women and highest in the group aged 60 - 79 years. *H. pylori* testing in a small minority of patients with GEJC (n = 5) showed a 60% positive rate; however, due to the small number of tested patients, no definitive conclusions can be drawn. The most frequent histologic type of GEJC was tubular adenocarcinoma. Only two patients with GEJC underwent IHC biomarker testing, limiting further conclusions.

In summary, we observed that there is a possible care gap in the adoption of precision medicine. The extremely low rates of guideline-recommended biomarker testing, even within this cohort of patients with a relatively high socioeconomic status and greater access to advanced medical services, is a critical observation. This finding highlights a profound and urgent need for an educational program for healthcare professionals in Brazil. The low rate of essential IHC biomarker testing, such as for HER2, among a cohort that, based on socioeconomic status, should have access to the highest standard of care highlights a significant gap in the implementation of modern oncology guidelines. Although the absence of PD-L1 and CLDN18.2 testing in patients with GEJC during the study period is understandable due to regulatory constraints, further investigation is needed to assess the prevalence of these biomarkers in the Brazilian population now that targeted therapies have been approved.

## Data Availability

All data generated or analyzed during this study, which support the findings of this study, are included within this article. Researchers may access analyses not present in the manuscript from the corresponding author upon reasonable request. The Astellas criteria on data sharing can be accessed via https://clinicalstudydatarequest.com/Study-Sponsors/Study-Sponsors-Astellas.aspx.

## Abbreviations

ANS: National Supplementary Health Agency
CLDN18: claudin 18
CLDN18.2: claudin 18.2
EBV: Epstein-Barr virus
EC: esophageal cancer
GC: gastric cancer
GEJ: gastroesophageal junction
GEJC: gastroesophageal junction cancer
*H. pylori*: *Helicobacter pylori*
HER2: human epidermal growth factor receptor 2
IHC: immunohistochemistry
IQR: interquartile range
MMR: mismatch repair
MSI: microsatellite instability
NET: neuroendocrine tumor
NOS: not otherwise specified
OS: overall survival
PD-L1: programmed cell death ligand 1
PFS: progression-free survival
UDE: upper digestive endoscopy
